# Correlation of ^68^Ga-FAPi-46 PET Biodistribution with FAP Expression by Immunohistochemistry in Patients with Solid Cancers: Interim Analysis of a Prospective Translational Exploratory Study

**DOI:** 10.2967/jnumed.121.262426

**Published:** 2022-07

**Authors:** Christine E. Mona, Matthias R. Benz, Firas Hikmat, Tristan R. Grogan, Katharina Lueckerath, Aria Razmaria, Rana Riahi, Roger Slavik, Mark D. Girgis, Giuseppe Carlucci, Kimberly A. Kelly, Samuel W. French, Johannes Czernin, David W. Dawson, Jeremie Calais

**Affiliations:** 1 Ahmanson Translational Theranostics Division, Department of Molecular and Medical Pharmacology, David Geffen School of Medicine, UCLA, Los Angeles, California;; 2 Jonsson Comprehensive Cancer Center, UCLA, Los Angeles, California;; 3 Institute of Urologic Oncology, UCLA, Los Angeles, California;; 4 Department of Medicine Statistics Core, David Geffen School of Medicine, UCLA, Los Angeles, California;; 5 Department of Pathology and Laboratory Medicine, David Geffen School of Medicine, UCLA, Los Angeles, California;; 6 Division of Surgical Oncology, Department of Surgery, David Geffen School of Medicine, UCLA, Los Angeles, California; and; 7 Department of Biomedical Engineering, University of Virginia School of Engineering and Applied Sciences, and Robert M. Berne Cardiovascular Research Center, University of Virginia School of Medicine, Charlottesville, Virginia

**Keywords:** cancer, PET/CT, fibroblast activation protein, immunohistochemistry, ^68^Ga-FAPi-46

## Abstract

Fibroblast activation protein (FAP)–expressing cancer-associated fibroblasts confer treatment resistance and promote metastasis and immunosuppression. Because FAP is overexpressed in many cancers, radiolabeled molecules targeting FAP are studied for their use as pancancer theranostic agents. This study aimed to establish the spectrum of FAP expression across various cancers by immunohistochemistry and to explore whether ^68^Ga FAP inhibitor (FAPi)–46 PET biodistribution faithfully reflects FAP expression from resected cancer and non-cancer specimens. **Methods:** We conducted a FAP expression screening using immunohistochemistry on a pancancer human tissue microarray (141 patients, 14 different types of cancer) and an interim analysis of a prospective exploratory imaging trial in cancer patients. Volunteer patients underwent 1 whole-body ^68^Ga-FAPi-46 PET/CT scan and, subsequently, surgical resection of their primary tumor or metastasis. ^68^Ga-FAPi-46 PET SUV_max_ and SUV_mean_ was correlated with FAP immunohistochemistry score in cancer and tumor-adjacent non-cancer tissues for each patient. **Results:** FAP was expressed across all 14 cancer types on tissue microarray with variable intensity and frequency, ranging from 25% to 100% (mean, 76.6% ± 25.3%). Strong FAP expression was observed in 50%–100% of cancers of the bile duct, bladder, colon, esophagus, stomach, lung, oropharynx, ovary, and pancreas. Fifteen patients with various cancer types (colorectal [*n* = 4], head and neck [*n* = 3], pancreas [*n* = 2], breast [*n* = 2], stomach [*n* = 1], esophagus [*n* = 2], and uterus [*n* = 1]) underwent surgery after their ^68^Ga-FAPi-46 PET/CT scan within a mean interval of 16.1 ± 14.4 d. ^68^Ga-FAPi-46 SUVs and immunohistochemistry scores were higher in cancer than in tumor-adjacent non-cancer tissue: mean SUV_max_ 7.7 versus 1.6 (*P* < 0.001), mean SUV_mean_ 6.2 versus 1.0 (*P* < 0.001), and mean FAP immunohistochemistry score 2.8 versus 0.9 (*P* < 0.001). FAP immunohistochemistry scores strongly correlated with ^68^Ga-FAPi 46 SUV_max_ and SUV_mean_: *r* = 0.781 (95% CI, 0.376–0.936; *P* < 0.001) and *r* = 0.783 (95% CI, 0.379–0.936; *P* < 0.001), respectively. **Conclusion:** In this interim analysis of a prospective exploratory imaging trial, ^68^Ga-FAPi-46 PET biodistribution across multiple cancers strongly correlated with FAP tissue expression. These findings support further exploration of FAPi PET as a pancancer imaging biomarker for FAP expression and as a stratification tool for FAP-targeted therapies.

## Visual Abstract

**Figure:**
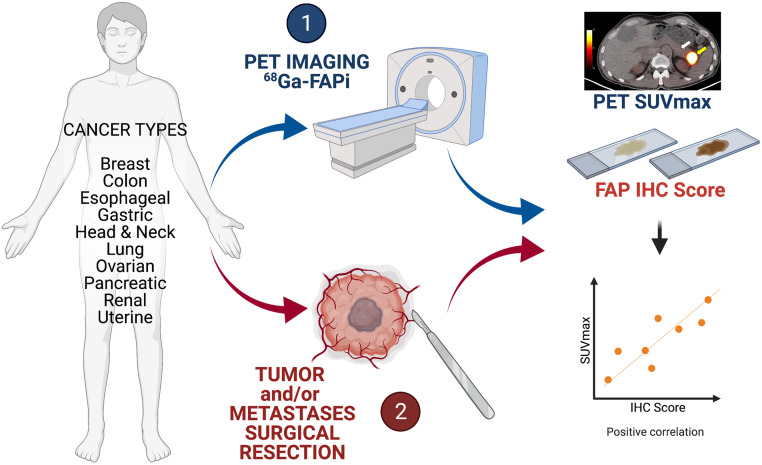

Fibroblast activation protein (FAP) is strongly expressed on cancer-associated fibroblasts and is a key player in tumor progression (*1*). High FAP expression is restricted almost exclusively to cancer-associated fibroblasts and serves as an independent negative prognostic factor for multiple types of cancer (*2*). In vivo depletion of FAP-positive stromal cells inhibits tumor growth by decreasing cancer support, increasing antitumor immunity, and limiting stromal barrier effects (*3*–*5*). However, targeting the enzymatic activity of FAP with antibodies does not yield beneficial clinical effects (*6**,**7*). Recently, FAP inhibitor (FAPi)–targeting ligands labeled with radioisotopes for PET imaging (e.g., ^68^Ga and ^18^F for PET) and therapy (e.g., ^177^Lu and ^90^Y) have been introduced (*8**,**9*). The high tumor uptake that was observed with FAPi PET imaging in various cancers suggests that radiolabeled FAPi compounds have promising potential for diagnostic and therapeutic applications (*10*).

In this prospective translational, exploratory study, we aimed at assessing the utility of FAPi PET imaging as a pancancer imaging biomarker for FAP expression. We first surveyed tissue microarrays (TMAs) of 141 patients with 14 cancer types for the presence and degree of FAP expression by immunohistochemistry (*11*). A cohort of surgical patients representing 10 of those cancer types was then tested to determine the correlation between ^68^Ga-FAPi-46 PET biodistribution and FAP immunohistochemistry expression in excised tumor tissue.

## MATERIALS AND METHODS

### TMA Screening

FAP expression in human tumor tissue was assessed using a pancancer TMA obtained from the University of Virginia. This TMA included 141 patients with 14 different types of cancer (bile duct, bladder, breast, colon, esophagus, stomach, liver, lung, ovary, oropharynx, pancreas, prostate, kidney, and uterus; 6–14 tumors per tissue type). Normal tissues present on the TMA were also evaluated (5–8 samples per tissue type). After deparaffinization and rehydration, heat-induced antigen retrieval (sodium citrate, 0.05% polysorbate 20, pH 6.0) was performed for 20 min using a vegetable steamer followed by quenching of endogenous peroxidase activity (3% hydrogen peroxide, 10 min). Primary antibody incubation with a 1:50 dilution of rabbit monoclonal anti-FAP α-[EPR20021] (ab207178; Abcam) was performed overnight at 4°C. Detection was performed using the ultraView Universal DAB Detection Kit (K3467; DAKO) per the manufacturer’s instructions. An experienced surgical pathologist (DWD) confirmed the histologic diagnoses and performed a immunohistochemistry analysis using a semiquantitative visual scoring system (0, negative staining; 1, weak staining; 2, strong staining).

### Clinical Study Design and Participants

We conducted a prospective exploratory biodistribution study of ^68^Ga-FAPI-46 PET imaging under the Radioactive Drug Research Committee Program (title 21 of *Code of Federal Regulations,* section 361.1). The primary objective was to define the biodistribution of ^68^Ga-FAPi-46 PET in normal and cancer tissues and further correlate with tissue expression as determined by FAP immunohistochemistry. Volunteer cancer patients scheduled to undergo surgical resection of a primary tumor or metastasis were eligible (the inclusion and exclusion criteria are in Supplemental Table 1; supplemental materials are available at http://jnm.snmjournals.org). The type of surgery depended on the location and disease as determined by clinical standard-of-care explorations. ^68^Ga-FAPi-46 PET/CT imaging findings did not impact the therapy plan, and surgery was performed independently of the results of the scan findings. The study was approved by the UCLA Institutional Review Board (approval 19-000756) and registered on ClinicalTrials.gov (NCT04147494). All patients provided oral and written informed consent.

We present here the results of an interim analysis that was mandated by the UCLA Jonsson Comprehensive Cancer Center Internal Scientific Peer Review Committee and Data Safety Monitoring Board after completed enrollment of 15 patients.

### FAPi PET/CT Image Acquisition

^68^Ga-FAPi-46 was used as the FAP-targeted radioligand (*8*). The mean injected activity was 184 ± 3 MBq (range, 174–185 MBq). The mean uptake time was 63 ± 10 min (range, 54–96 min). Images were acquired using 64-detector PET/CT scanners (Biograph 64 mCT [*n* = 7] or Biograph 64 TruePoint [*n* = 8]; Siemens Healthcare). Unenhanced CT (120 kV, 80 mAs) was performed for attenuation correction and anatomic correlation of the PET findings. PET images were acquired from vertex to mid thigh, using an emission time of 2–4 min per bed position, depending on patient body weight. All PET images were reconstructed using correction for attenuation, dead time, random events, and scatter. PET images were reconstructed using an iterative algorithm (ordered-subset expectation maximization).

### FAPi PET/CT Image Analysis

Images were analyzed in consensus by 2 readers (MRB, JCa) blinded to the histopathology and immunohistochemistry results. The readers had access to all medical records and other imaging modality results available to facilitate tumor localization. Image analysis was performed with OsiriX (Pixmeo) (*12*). The readers quantified the ^68^Ga-FAPi-46 PET uptake in cancer tissue and tumor-adjacent non-cancer tissue by placing volumes of interest in the tumor lesions and the surrounding normal tissue in the same organ. The readers adapted the size of the volume of interest visually to best encompass the structure of interest and to preclude overlapping of activity between the cancer and non-cancer volumes of interest. Anatomic CT information was used to avoid activity spillover from other organs. SUV_mean_, SUV_max_, and lesion size by CT were recorded.

### Histopathology and Immunohistochemistry Analysis

Clinical pathology reports were used to collect final pathology diagnoses and pathologic TNM staging. Representative sections of normal and tumor tissue from surgical resection specimens were obtained from the UCLA Department of Pathology through the UCLA Translational Pathology Core Laboratory. FAP immunohistochemistry staining was performed as described above.

All hematoxylin and eosin slides from each surgical pathology case were evaluated to select representative sections consisting of normal and tumor tissue for immunohistochemistry evaluation. One representative section that best reflected the overall tumor histology (i.e., histologic type and grade, relative stroma and tumor cell component), that included sampling of both the edge and the central portions of the tumor mass, and that contained surrounding adjacent normal tissue (>5 mm distance from malignant cells) was selected for each patient. Immunohistochemistry stains were independently scored by 2 pathologists (DWD, SWF) who did not know each other’s scores, the clinical information, or the PET imaging results. A semiquantitative approach adapted from a prior study was used (*13*). Briefly, FAP expression was assessed globally across the entire cross-sectional area of tumor and adjacent nonmalignant tissue without any specific focus on invasive fronts or areas of active tumor growth. The tumor compartment was defined on the basis of morphologic assessments as the geographic area where malignant cells were present, as well as the immediately adjacent area of intratumoral and peritumoral stromal response. A score of 0 was defined as complete absence of staining or weak staining in less than 10% of the area under assessment. A score of 1 was defined as weak expression in greater than 10% of the area under assessment. A score of 2 was defined as moderate or strong expression in 10%–50% of the area under assessment. A score of 3 was defined as moderate or strong expression in more than 50% of the area under assessment.

### Cross-Sectional Correlation Analysis of the FAPi PET Signal and FAP Immunohistochemistry Staining

The ^68^Ga-FAPi-46 PET SUV and the FAP immunohistochemistry score of cancer and tumor-adjacent non-cancer tissue were evaluated for correlation on a per-patient basis: for each tumor lesion, the ^68^Ga-FAPi-46 PET SUV of the lesion was evaluated for correlation with the immunohistochemistry score of the tumor compartment on the selected pathology slide, and the ^68^Ga-FAPi-46 PET SUV of the normal tissue surrounding the tumor lesion was evaluated for correlation with the immunohistochemistry score of the tumor-adjacent non-cancer tissue available on the same pathology slide as that containing the tumor lesion.

### Statistics

Patient characteristics and study variables were summarized using mean, SD, ranges, or frequency (%) as appropriate. To test for differences in expression levels of both immunohistochemistry and PET measures between cancer and non-cancer tissues, the 2 groups were compared using *P* values from a generalized-estimating-equation model (to properly account for the repeated-measures design of the study) (*14*). For assessing the association between immunohistochemistry and PET findings, we computed repeated-measures correlation coefficients. Interreader agreement for the immunohistochemistry scoring was assessed using Cohen κ-statistics. *P* values of less than 0.05 were considered statistically significant. Analyses were performed using SAS (version 0.4; SAS Institute) and R (version 3.6.1, Rmcorr package; www.r-project.org). Because of the exploratory nature of this study and the Radioactive Drug Research Committee–mandated limit of 30 patients, with an interim analysis after 15 patients mandated by the UCLA Institutional Review Board, a power analysis for sample size was not performed.

## RESULTS

### TMA Analysis

**FIGURE 1.**
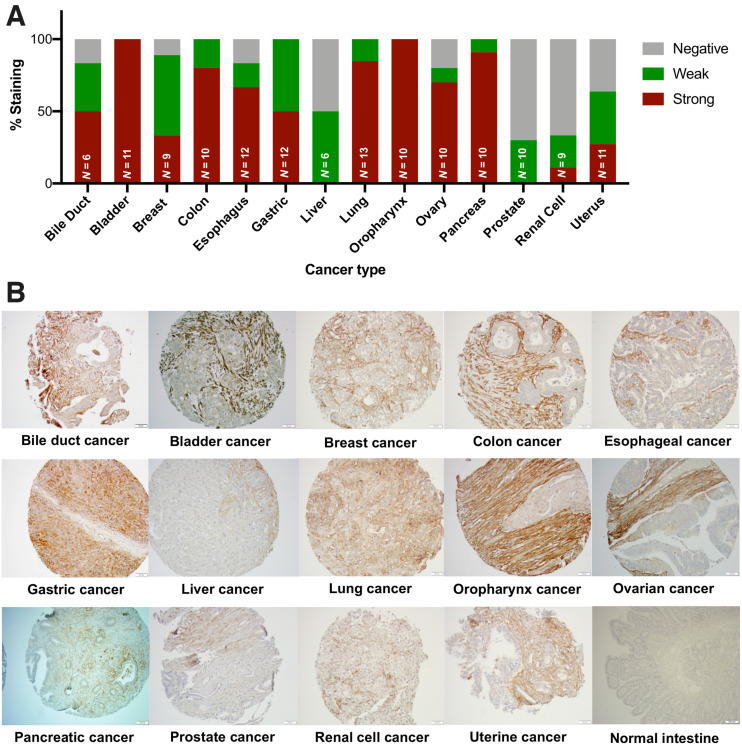
FAP expression by immunohistochemistry in 14 cancer types and normal tissues (TMA analysis). (A) Quantification of FAP expression per cancer type. FAP intensity was evaluated using semiquantitative visual scoring system that accounts for staining intensity (0, negative; 1, weak; 2, strong). (B) FAP immunohistochemistry expression on representative tissue core from indicated cancer or normal tissue type.

Representative FAP immunohistochemistry scoring by cancer type performed in the TMA is shown in Figure 1. FAP expression was present in 80.9% (114/141) of tumors. Of the 114 positive tumors, FAP expression was stromal in 108, epithelial in 1, and mixed in 5 (lung cancer [*n* = 1], ovarian cancer [*n* = 1], oropharynx [*n* = 1], pancreatic [*n* = 1], and uterine cancer [*n* = 1]). No stroma was present for evaluation in 1 case of ovarian cancer (0.7%).

Although there was variability in the intensity and frequency of FAP expression, FAP was positive in more than 50% of cases from 11 of 14 cancer types. Strong FAP expression was observed in 50%–100% of cancers from the bile duct, bladder, colon, esophagus, stomach, lung, oropharynx, ovary, and pancreas. Liver, prostate, and renal cell cancer were the 3 tumor types with the lowest FAP expression.

This TMA survey provided a rationale for the design of the subsequent clinical PET imaging study.

### PET Imaging Study Cohort

Between December 2019 and May 2020, 15 patients (8 men and 7 women; mean age, 60.7 ± 10.5 y) with 7 different cancer types (colorectal [*n* = 4], head and neck [*n* = 3], pancreatic [*n* = 2], breast [*n* = 2], gastric [*n* = 1], esophageal [*n* = 2], and uterine [*n* = 1] cancer) were enrolled. Supplemental Table 2 summarizes the demographics and clinical characteristics of the study population. All 15 patients underwent ^68^Ga-FAPi-46 PET/CT and subsequent surgery within 16.1 ± 14.4 d (range, 1–50 d) after the scan. Two patients had tumors deemed unresectable at the time of surgery (gastric linitis plastica with duodenal extension, patient 3; pancreatic cancer with venous involvement, patient 14).

### ^68^Ga-FAPi-46 PET Biodistribution in Cancer Lesions, Normal Organs, and Non-Cancer Tissues

The ^68^Ga-FAPi-46 biodistribution as determined by SUV_mean_ in normal organs is described in Supplemental Table 3 and Supplemental Figure 1. The ^68^Ga-FAPi-46 SUVs and the size of the cancer lesions are provided in Supplemental Tables 4 (primary tumors) and 5 (metastases).

#### Normal Organs and Non-Cancer Tissues

The highest normal-organ ^68^Ga-FAPi-46 PET signals were in the urinary bladder (because of urinary excretion) and the uterus (because of normal myometrial FAP expression). Other organs with notable ^68^Ga-FAPi-46 uptake included the submandibular glands, Waldeyer ring, pancreas, and kidneys (average SUV_mean_ < 2.5). ^68^Ga-FAPi-46 uptake higher than in normal tissues was noted in 3 lesions (SUV_max_ of 4.4, 2.4, and 2.6) that subsequently revealed a benign pathology, including an elastofibroma dorsi (patient 3) and 2 areas of fibrosis or scarring in breast tissue (patient 11).

#### Cancer Tissues

The average ^68^Ga-FAPi-46 SUV_mean_ and SUV_max_ was 7.2 ± 4.4 (range, 1.5–15.2) and 8.6 ± 5.2 (range, 1.7–19), respectively, in primary tumors (*n* = 15) and 4.3 ± 2.9 (range, 2.1–8.8) and 5.3 ± 3.6 (range, 2.7–10.8), respectively, in metastases (*n* = 6). The cancer types with the highest uptake were those of the pancreas, stomach, colon, and uterus. The lowest uptake was in 2 patients with a complete response to neoadjuvant therapy (patients 13 and 15) and thus low FAP expression

### Immunohistochemistry Findings

Histologic sections from 13 patients who underwent tumor resection were analyzed. Normal tissue adjacent to tumors and tumor tissue from individual histologic sections were available for immunohistochemistry in 13 of 15 (87%) and 11 of 15 patients (73%), respectively. Primary tumor, metastasis, or both primary tumor and metastasis were evaluated in 7 of 11 (63%), 2 of 11 (18%), and 2 of 11 (18%) cases, respectively. The FAP scoring between the 2 pathologists was in almost perfect agreement (κ = 0.89).

#### Primary Tumors

**FIGURE 2.**
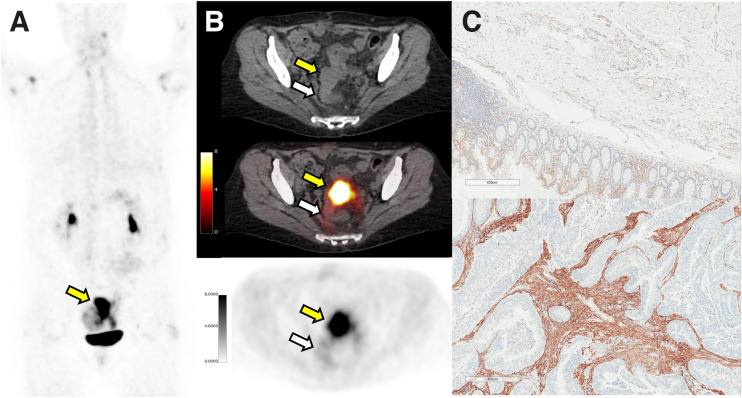
Matched ^68^Ga-FAPI-46 PET/CT and immunohistochemistry results for patient 10, 56-y-old woman with sigmoid colon adenocarcinoma who underwent colorectal anterior resection (ypT4b N0 M0). In area corresponding to resected mass as shown by yellow arrows (PET maximum-intensity projection [A], axial CT [B, top], axial PET/CT [B, middle], and axial PET [B, bottom]), ^68^Ga-FAPi-46 PET/CT showed intense uptake (SUV_max_, 15.9; SUV_mean_, 12.8). FAP immunohistochemistry on representative histologic sections demonstrated absent to weak FAP expression seen predominantly as vessel endothelial cell staining in normal tissue (C, top) and strong FAP expression in intratumoral and peritumoral stromal (C, bottom). White arrows depict normal region resected.

The highest FAP immunohistochemistry scores were observed in pancreatic, esophageal, and breast cancer. FAP staining was confined exclusively to the tumor-associated stromal compartment in most patients (12/13; 92.3%) and ranged from weak to strong expression (*1*–*3*). The staining intensity was the greatest in stromal areas within and immediately adjacent to (peritumoral) the malignant epithelial compartment of tumors as shown in a case example in Figure 2 (patient 10).

#### Metastatic Lesions

All 4 evaluated metastatic lesions (3 lymph nodes and 1 liver metastasis) were positive for FAP, including stromal staining in 3 of the 4 and malignant epithelial cell staining in 1 of the 4 (uterine squamous cell carcinoma involving a left pelvic lymph node, patient 8). FAP staining was equivalent between primary and metastatic lesions in 2 patients with tissue available for comparative analysis (patients 6 and 15, Supplemental Fig. 2).

#### Tumor-Adjacent Non-Cancer Tissues

Staining was absent or weak in most normal tissues (71.4% negative, 25% weak, 3.6% moderate) and observed primarily in capillary and small-vessel endothelium. FAP expression was moderate in a concurrently resected benign elastofibroma dorsi (patient 3) and was moderate to strong in 2 areas of radial scarring and biopsy site changes in benign breast tissue without cancer (patient 11, Supplemental Fig. 3).

### Correlation of ^68^Ga-FAPi-46 PET Signal and FAP Immunohistochemistry Staining in Cancer and Tumor-Adjacent Non-Cancer Tissues (Per-Patient Analysis)

Supplemental Figures 4–16 depict each patient case with an available cross-sectional correlation analysis of the ^68^Ga-FAPi-46 PET signal and FAP immunohistochemistry staining score.

**FIGURE 3.**
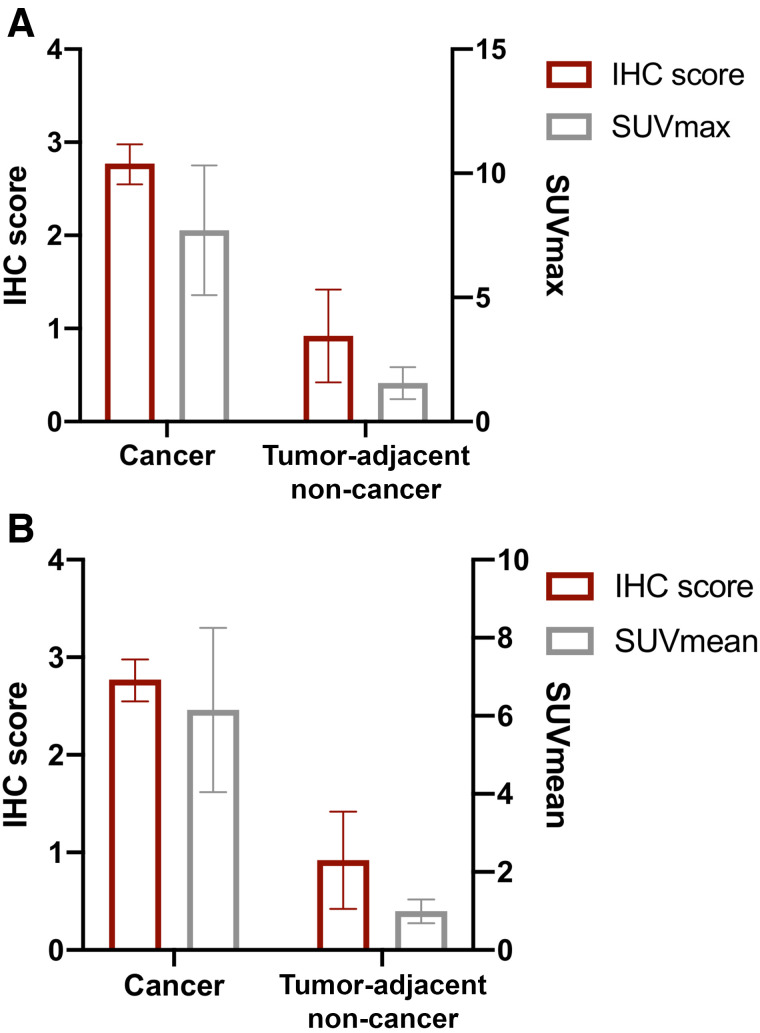
FAP immunohistochemistry (IHC) score with ^68^Ga-FAPi-46 PET SUV_max_ (A) and SUV_mean_ (B) in cancer and tumor-adjacent non-cancer tissues. Each bar represents mean with SD.

^68^Ga-FAPi-46 SUV_max_ and SUV_mean_, and the FAP immunohistochemistry score, were higher in cancer tissue than in tumor-adjacent non-cancer tissue: mean SUV_max_ was 7.7 (95% CI, 5.1–10.3) versus 1.6 (95% CI, 0.9–2.2; *P* < 0.001), respectively; mean SUV_mean_ was 6.2 (95% CI, 4.0–8.3) versus 1.0 (95% CI, 0.7–1.3; *P* < 0.001), respectively; and mean FAP immunohistochemistry score was 2.8 (95% CI, 2.6–3.0; *P* < 0.001) versus 0.9 (95% CI, 0.4–1.4; *P* < 0.001), respectively (Fig. 3).

**FIGURE 4.**
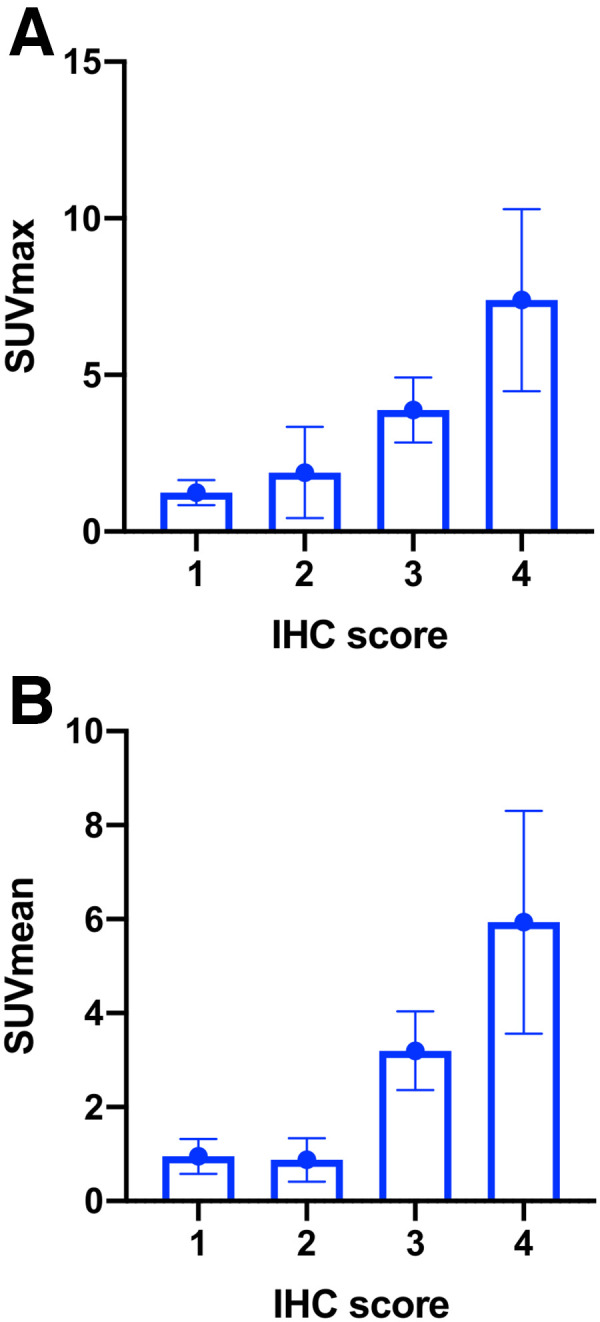
Correlation between FAP immunohistochemistry (IHC) score and ^68^Ga-FAPi-46 PET SUV_max_ (A) and SUV_mean_ (B) across cancer and tumor-adjacent non-cancer tissues. *r* = 0.781 in A (95% CI, 0.376–0.936; *P* < 0.001) and *r* = 0.783 in B (95% CI, 0.379–0.936; *P* < 0.001).

The FAP immunohistochemistry score correlated positively both with ^68^Ga-FAPi-46 SUV_max_ across cancer and tumor-adjacent non-cancer tissues (*r* = 0.781 [95% CI, 0.376–0.936], *P* < 0.001) and with SUV_mean_ (*r* = 0.783 [95% CI, 0.379–0.936], *P* < 0.001) (Fig. 4). FAP immunohistochemistry scores of 0, 1, 2, and 3 corresponded to a mean ^68^Ga-FAPi-46 SUV_max_ of 1.2 (95% CI, 0.8–1.6), 1.9 (95% CI, 0.4–3.3), 3.9 (95% CI, 2.8–4.9), and 7.4 (95% CI, 4.5–10.3), respectively. CT size tended to correlate positively with SUV_max_ (Spearman *r* = 0.57; *P* = 0.054) and SUV_mean_ (Spearman *r* = 0.54; *P* = 0.068).

## DISCUSSION

In this translational study, we aimed to establish the spectrum of FAP expression across various cancers by immunohistochemistry and to explore whether ^68^Ga-FAPi-46 PET biodistribution faithfully reflects FAP expression in cancer patients. We report here the results of a TMA analysis from 141 patients with 14 different types of cancer and of an interim analysis of a prospective exploratory imaging trial that included 15 patients. FAP was expressed across all cancer types with variable intensity and frequency. We established a positive and significant correlation between FAP-target expression and FAPi PET SUVs.

Cancer-associated fibroblasts are key constituents of the tumor stroma that can support an immunosuppressive microenvironment and tumor cell growth, progression, and metastatic potential (*1*). Depleting the stroma can improve delivery of drugs or systemically applied radiation and enhance cancer immune responses (*15*). Thus, FAP expressed by cancer-associated fibroblasts is an attractive diagnostic and therapeutic target (*16*). Target specificity and tumor-specific uptake are critical determinants of the accuracy and efficacy of PET probes for diagnosis and therapy (*17*). FAP frequently is strongly expressed in solid tumors, with only limited expression in normal tissues, making it an attractive theranostic target (*10*).

FAPi PET imaging has reported high tumor-to-background characteristics (*10*). However, FAPi PET human biodistribution in cancer has not been validated against tumor FAP expression as assessed by immunohistochemistry in a pancancer approach. Recently, a study showed a strong association between tumor ^68^Ga-FAPi-46 PET uptake intensity and histopathologic FAP expression in sarcoma tumors (*18*). Here, we first screened TMAs from 14 cancers for FAP expression to guide patient selection for the exploratory imaging trial. Guided by our initial TMA screening, we intentionally selected multiple cancer types to validate the pancancer approach. In the interim analysis of this prospective exploratory trial, the biodistribution of ^68^Ga-FAPi-46 PET correlated strongly with FAP expression in cancer versus normal tissues across 7 different cancer types, supporting its potential role as a pancancer predictive biomarker for FAP-targeted therapies. In a subset of patients, the ^68^Ga-FAPi-46 SUV_max_ of metastasis was also comparable to that of their primary tumor, suggesting that FAP expression may be consistent across primary and metastatic lesions within individual patients, which has important implications for its role as a theranostic in the setting of advanced disease (*19*).

These findings support further exploration of ^68^Ga-FAPi-46 as a potential pancancer imaging biomarker for FAP expression. This use could find application as an enrichment biomarker or patient selection tool for clinical trials and as a potential predictor of treatment response in the clinic. Extensive emerging data implicate FAP-positive cells as important accomplices involved in cancer progression and metastases. Evaluating FAP-targeting small-molecule inhibitors, antibodies, bispecific T-cell engagers, and radioligand therapy requires a means for verifying whole-body target expression (*20*).

The main limitation of the study was the small sample size. This was an exploratory study, and local oversight committees (Internal Scientific Peer Review Committee, Data Safety Monitoring Board) mandated an interim analysis after the first 15 patients. This interim analysis revealed a strong correlation between immunohistochemistry and PET findings in 14 patients, which provided the motivation to publish the data.

Another major limitation was the intratumor heterogeneity and sampling bias inherent in the histopathology and immunochemistry analysis. Unfortunately, autoradiography was not possible in this exploratory study because a second administration of ^68^Ga-FAPi-46 just before surgery was not practical. We performed an evaluation of all hematoxylin and eosin slides from each surgical pathology case to select the section best representing the overall tumor histology or its surrounding tumor-adjacent non-cancer tissue.

A perfect anatomic match between tumor SUV measurements and immunohistochemistry scores was unfortunately not possible because tumors were not resected in a defined orientation (unlike in prostate cancer). Therefore, we collected the SUV_max_ and SUV_mean_ of the whole tumor lesion.

Another limitation is that visual immunohistochemistry scoring by pathologists is semiquantitative only, is subjective, and produces ordinal rather than continuous variable data. Computer-aided analysis with automatic immunohistochemistry scoring may overcome these limitations. However, even with semiquantitative ordinal data, the correlation of immunohistochemistry scoring with SUV was strong. Furthermore, the interreader scores between the 2 pathologists was in near-perfect agreement.

## CONCLUSION

In this interim analysis of a prospective exploratory imaging trial, ^68^Ga-FAPi-46 PET biodistribution correlated strongly with FAP expression in cancer and tumor-adjacent non-cancer tissues across multiple cancer types. These data support the use of ^68^Ga-FAPi-46 PET as a pancancer predictive biomarker and stratification tool for FAP-targeted therapeutic approaches and lay the foundation for future evaluation of FAPi ligands labeled with therapeutic isotopes in clinical trials.

## Notes

KEY POINTS **QUESTION:** Is FAPi PET imaging a reliable biomarker of FAP expression in cancer and tumor-adjacent non-cancer tissues? **PERTINENT FINDINGS:** In this translational study using TMA and an interim analysis of a prospective exploratory imaging trial in 15 surgical oncology patients, the FAPi PET uptake and FAP expression per immunohistochemistry correlated strongly in cancer and tumor-adjacent non-cancer tissue. **IMPLICATIONS FOR PATIENT CARE:** FAPi PET uptake correlates strongly with FAP expression in cancer patients, and FAPi PET may thus serve as a predictive biomarker for FAP-targeted therapeutic approaches.

## References

[1] Gascard P Tlsty TD. Carcinoma-associated fibroblasts: orchestrating the composition of malignancy. Genes Dev. 2016;30:1002–1019. 27151975 10.1101/gad.279737.116 PMC4863733

[2] Fitzgerald AA Weiner LM. The role of fibroblast activation protein in health and malignancy. Cancer Metastasis Rev. 2020;39:783–803. 32601975 10.1007/s10555-020-09909-3 PMC7487063

[3] Lo A Wang LS Scholler J. Tumor-promoting desmoplasia is disrupted by depleting FAP-expressing stromal cells. Cancer Res. 2015;75:2800–2810. 25979873 10.1158/0008-5472.CAN-14-3041 PMC4506263

[4] Fearon DT. The carcinoma-associated fibroblast expressing fibroblast activation protein and escape from immune surveillance. Cancer Immunol Res. 2014;2:187–193. 24778314 10.1158/2326-6066.CIR-14-0002

[5] Kraman M Bambrough PJ Arnold JN. Suppression of antitumor immunity by stromal cells expressing fibroblast activation protein-alpha. Science. 2010;330:827–830. 21051638 10.1126/science.1195300

[6] Eager RM Cunningham CC Senzer N. Phase II trial of talabostat and docetaxel in advanced non-small cell lung cancer. Clin Oncol (R Coll Radiol). 2009;21:464–472. 19501491 10.1016/j.clon.2009.04.007

[7] Liu R Li H Liu L Yu J Ren X. Fibroblast activation protein: a potential therapeutic target in cancer. Cancer Biol Ther. 2012;13:123–129. 22236832 10.4161/cbt.13.3.18696

[8] Loktev A Lindner T Burger EM. Development of fibroblast activation protein-targeted radiotracers with improved tumor retention. J Nucl Med. 2019;60:1421–1429. 30850501 10.2967/jnumed.118.224469 PMC6785792

[9] Loktev A Lindner T Mier W. A tumor-imaging method targeting cancer-associated fibroblasts. J Nucl Med. 2018;59:1423–1429. 29626120 10.2967/jnumed.118.210435 PMC6126438

[10] Kratochwil C Flechsig P Lindner T. ^68^Ga-FAPI PET/CT: tracer uptake in 28 different kinds of cancer. J Nucl Med. 2019;60:801–805. 30954939 10.2967/jnumed.119.227967 PMC6581228

[11] Kallioniemi OP Wagner U Kononen J Sauter G. Tissue microarray technology for high-throughput molecular profiling of cancer. Hum Mol Genet. 2001;10:657–662. 11257096 10.1093/hmg/10.7.657

[12] Rosset A Spadola L Ratib O. OsiriX: an open-source software for navigating in multidimensional DICOM images. J Digit Imaging. 2004;17:205–216. 15534753 10.1007/s10278-004-1014-6 PMC3046608

[13] Henry LR Lee HO Lee JS. Clinical implications of fibroblast activation protein in patients with colon cancer. Clin Cancer Res. 2007;13:1736–1741. 17363526 10.1158/1078-0432.CCR-06-1746

[14] Bakdash JZ Marusich LR. Repeated measures correlation. Front Psychol. 2017;8:456. 28439244 10.3389/fpsyg.2017.00456 PMC5383908

[15] Barsoumian HB Ramapriyan R Younes AI. Low-dose radiation treatment enhances systemic antitumor immune responses by overcoming the inhibitory stroma. J Immunother Cancer. 2020;8:e000537. 33106386 10.1136/jitc-2020-000537 PMC7592253

[16] Narunsky L Oren R Bochner F Neeman M. Imaging aspects of the tumor stroma with therapeutic implications. Pharmacol Ther. 2014;141:192–208. 24134903 10.1016/j.pharmthera.2013.10.003 PMC3947248

[17] Huang R Wang M Zhu Y Conti PS Chen K. Development of PET probes for cancer imaging. Curr Top Med Chem. 2015;15:795–819. 25732787 10.2174/1568026615666150302110325

[18] Kessler L Ferdinandus J Hirmas N. ^68^Ga-FAPI as a diagnostic tool in sarcoma: data from the ^68^Ga-FAPI PET prospective observational trial. J Nucl Med. 2022;63:89–95. 33931468 10.2967/jnumed.121.262096 PMC8717183

[19] Arranja AG Pathak V Lammers T Shi Y. Tumor-targeted nanomedicines for cancer theranostics. Pharmacol Res. 2017;115:87–95. 27865762 10.1016/j.phrs.2016.11.014 PMC5412956

[20] Lindner T Loktev A Giesel F Kratochwil C Altmann A Haberkorn U. Targeting of activated fibroblasts for imaging and therapy. EJNMMI Radiopharm Chem. 2019;4:16. 31659499 10.1186/s41181-019-0069-0 PMC6658625

